# Circulating tumor DNA in diffuse large B-cell lymphoma: analysis of response assessment, correlation with PET/CT and clone evolution

**DOI:** 10.1016/j.htct.2024.07.005

**Published:** 2024-09-20

**Authors:** Guilherme Duffles, Jersey Heitor da Silva Maués, Fernanda Lupinacci, Luciana Guilhermino Pereira, Elisa Napolitano Ferreira, Leandro Freitas, Fernanda Niemann, Maria Emilia Seren Takahashi, Celso Darío Ramos, Maria de Lourdes L. Ferrari Chauffaille, Irene Lorand-Metze

**Affiliations:** a University of Campinas, Hematology and Hemotherapy Centre, Hematology, Unicamp, Campinas 13083-878, SP, Brazil; b Rede Dor Sao Luiz, Sao Paulo 01401-002, SP, Brazil; c Fleury Medicina e Saúde, Grupo Fleury, São Paulo 04580-060, SP, Brazil; d Department of Pathology, School of Medical Sciences, University of Campinas (UNICAMP), Campinas, 13083-888, SP, Brazil; e Gleb Wataghin Institute of Physics, University of Campinas (UNICAMP), Campinas 13083-859, SP, Brazil; f Division of Nuclear Medicine, School of Medical Sciences, University of Campinas (UNICAMP), Campinas 13083-888, SP, Brazil

**Keywords:** ctDNA, Liquid biopsy, Diffuse large B-cell lymphoma (DLBCL), Lymphoma, Clone evolution, PET/CT, Radiomics

## Abstract

**Introduction:**

Circulating tumor DNA (ctDNA) can be obtained from cell-free DNA (cfDNA) andis a new technique for genotyping, response assessment and prognosis in lymphoma.

**Methods:**

Eighteen patients with samples at diagnosis (ctDNA1), after treatment (ctDNA2) and extracted from diagnostic tissue (FFPE) were evaluated.

**Results:**

In all patients, at least one mutation in cfDNA was detected at diagnosis. *CREBBP* was the most frequent mutated gene (67 %). In 12 of the 15 patients with complete remission, the mutation attributed to the disease found at diagnosis cleared with treatment. A reduction in the ctDNA was observed after treatment in 14 patients, 12 of whom achieved complete remission. Correlations were found between the ctDNA at diagnosis and total metabolic tumor volume (*r* = 0.51; *p*-value = 0.014) and total lesion glycolysis 2.5 (*r* = 0.47; *p*-value = 0.024) by PET at diagnosis and between ctDNA at diagnosis and radiomic features of the lesions with the largest standardized uptake value. There was a strong inverse correlation between ΔctDNA1 and ΔSUVmax by PET/CT (*r* = −0.8788; *p*-value = 0.002).

**Conclusion:**

Analysis of ctDNA and PET/CT in large B-cell lymphoma are complementary data for evaluating tumor burden and tumor clearance after treatment. Analysis of radiomic data might help to identify tumor characteristics and their changes after treatment.

## Introduction

Diffuse large B-cell lymphoma (DLBCL) is the most common type of lymphoma, with a cure rate of 60 % with standard immunochemotherapy.^1^^,^^2^ Patients with refractory DLBCL have a dismal prognosis when treated with conventional salvage chemotherapy, with a median overall survival of about seven months.^3^ Therefore, laboratory tests that are sensitive enough to detect the status of tumor activity are useful.

Cell-free DNA (cfDNA) are fragments of DNA circulating in peripheral blood derived from cells after apoptosis or necrosis, mainly leukocytes and endothelial cells.^4^^,^^5^ In patients with neoplasms, a part of the cfDNA is composed of circulating tumor DNA (ctDNA).^6^^,^^7^ The amount of ctDNA is proportional to the disease burden and histological type.^7^^,^^8^ In recent years, several studies have shown that the evaluation of the ctDNA in peripheral blood at diagnosis, as a non-invasive procedure, is useful to measure the tumor burden, analyze the molecular profile and monitor treatment of lymphomas.^9^, ^10^, ^11^, ^12^, ^13^ Moreover, it may be a better test to detect relapse compared to standard imaging procedures.^11^^,^^14^ Different from a tissue biopsy, which analyses data from a specific tumor site, ctDNA allows assessment of spatial tumor heterogeneity and may be able to characterize the complete mutational profile of the patient's malignancy.^11^^,^^15^ Its prognostic importance in DLBCL has also been demonstrated.^11^^,^^16^, ^17^, ^18^

On the other hand, new positron emission tomography (PET)/computed tomography (CT) quantitative measures have been developed with the potential of better quantifying tumor metabolic burden. Total metabolic tumor volume (TMTV), that is, the sum of the metabolic volumes of each of the lesions and total lesion glycolysis (TLG), that is, TMTV x average standardized uptake value (SUV) of the segmented tumor volume, present a high correlation with the total tumor burden.^19^ The prognostic role of TMTV has been described including in a randomized clinical trial.^20^^,^^21^ The utility of combining ctDNA and PET/CT has been explored, with correlation of these different parameters as measures of tumor burden.^18^^,^^22^, ^23^, ^24^ Another new development of PET analysis concerns radiomics, where biomarkers of the disease can be quantified.^25^^,^^26^ This measure, associated with other biological information of aggressive lymphomas, can aid in the prognostic evaluation.^27^ Combining ctDNA with various prognostic parameters has been shown in DLBCL, with dynamic evaluation during treatment.^28^

Clonal evolution involves complex interplays of passenger and driver mutations, with direct impact of treatment.^29^ This has been analyzed in DLBCL, but mainly in relapse/refractory cases using diagnostic tissue.^30^^,^^31^ ctDNA is a possible material for studying tumor clonal evolution but this has rarely been done in lymphoma.^32^

The present study shows an analysis of ctDNA collected at diagnosis and at the end of treatment from DLBCL patients of a Brazilian reference center, using next-generation sequencing (NGS) with a predetermined panel of genes. It also compares ctDNA and PET/CT and performs a clone evolution strategy in a small subset of patients.

## Materials and methods

### Study design

Consecutive newly diagnosed patients with DLBCL attended at our institution, a Hematology Reference Center in the southeastern region of Brazil, between October 2018 and November 2021 were studied. Diagnosis of DLBCL was based on the current WHO classification on lymphoid neoplasms,^33^ with determination of cell-of-origin using the Hans algorithm.^34^ Patients with existing neoplasms (including any type of lymphoma), history of chemotherapy or organ transplantation, renal failure, hepatic dysfunction or presence of active infection from the HIV virus, hepatitis C or hepatitis B were excluded. All patients read and signed the informed consent form. This study was planned and conducted according to the Declaration of Helsinki and was approved by the local research ethics committee (Proc. 87316418.7.0000.5404).

Blood samples for plasma cell-free DNA examination before treatment (cfDNA1) were collected in a specific DNA-stabilizing tube (Cell-Free DNA BCT - Streck®) and processed within five days of collection. ctDNA was extracted from cfDNA. Genomic DNA from the diagnostic biopsy using formalin-fixed paraffin-embedded (FFPE) blocks was also analyzed. Eight weeks after the last chemotherapy, a second peripheral blood sample was collected for cfDNA analysis (cfDNA2). Only patients with the three samples available (FFPE, circulating tumor DNA at diagnosis [ctDNA1] and after treatment [ctDNA2]) were included in the analysis.

Staging was achieved with PET/CT or CT of the neck, chest, and abdomen. Bone marrow biopsy was mandatory when the PET was not performed. Patients received standard treatment with R-CHOP (rituximab, cyclophosphamide, doxorubicin, prednisone, and vincristine) Primary gastrointestinal (GI) lymphoma was staged according to Lugano GI tract classification system.^35^ Response assessment and follow-up were performed according to the Lugano recommendation.^36^ Non-responding patients were treated based on the institutional protocol for salvage therapy.

### PET/CT analysis

The volumetric parameters, TMTV and TLG, were calculated from the initial whole-body ^18^F-FDG PET/CT images of the study subjects. Radiomic features were based on textural features extracted from the gray-tone spatial-dependence matrices as proposed by Haralick et al.^37^ For patients who underwent PET/CT imaging at diagnosis and at the end of treatment, the difference (Δ) was computed between the PET parameters of the two images. For instance, ΔSUVmax was calculated as (SUVmax1 – SUVmax2). The same approach was applied for TMTV and TLG. Please see supplemental text for more details.

### DNA extraction, library preparation and ctDNA quantification

DNA was extracted from plasma (cfDNA) and tissue (FFPE) using the ThermoFisher MagMAX kit, according to the user guide.^38^ The Qubit 2.0 flurometer (Thermo Fisher) was used for the cfDNA concentration. The QIAseq Targeted DNA Panel (QIAGEN), which covers the coding regions of 11 genes (*KMT2D, LRP1B, HIST1H1E, PIM1, PCLO, TP53, CARD11, CREBBP, MYD88, CD79B, B2M*) was used in the preparation of the library. This panel was customized for this study based on data of frequently mutated genes in DLBCL.^39^ The Ion Torrent S5 (Ion 520/530 Chip Kit for PGM and S5 Prime) was used for next-generation sequencing.

The product of cfDNA quantification and mean value of the variant allele frequency (VAF) of somatic mutations in genome equivalents per milliliter (hGE/mL), were used to measure the ctDNA, as a quantitative value, and a log10 scale was calculated for the difference between ctDNA1 and ctDNA2, as previously described.^16^ See the supplemental text for the genomic analysis and clone evolution.

### Statistical analysis

The Statistical Package for Social Sciences (SPSS) version 22 was used for clinical data analysis. Due to the small number of cases studied, only nonparametric tests were used**.** Quantitative variables are presented as median and range (minimum and maximum values). Categorical variables are described with counts and proportions. Linear regression was calculated with the ggpubr package and was considered significant for *p*-values <0.05. All statistics for genomic data were performed using R (https://www.r-project.org).

The association of features of the genomic analysis and radiomic parameters obtained in PET/CT at diagnosis were also analyzed. Additionally, a multiple regression was performed to examine the best radiomic variables associated with ctDNA1 using those showing a correlation in the Kruskal–Wallis test with *p*-values <0.05 for input and *p*-values <0.10 for output using the backward conditional method.

## Results

Between October 2018 and November 2021, 27 patients with newly diagnosed DLBCL collected peripheral blood for cfDNA analysis at diagnosis. In four cases, there was a lack of viable tissue for DNA extraction (FFPE) and in five patients the cfDNA2 was not collected as four patients had died during treatment due to infections and one due to progressive disease. Therefore, a complete analysis of FFPE, cfDNA1 and cfDNA2 was possible in only 18 patients. Table 1 shows the clinical characteristics of patients.

**Table 1:** Clinical and treatment characteristics.

**Characteristic**	**Patients**
Age in years - median (range)	59.5 (24–84)
Sex - n (%)	
Male	8 (44.4)
Female	10 (55.6)
Bulky disease - n (%)	
Present	6 (33.3)
Absent	12 (66.7)
IPI (NCCN) - n (%)	
Low (0 or 1)	2 (11.1)
Low-intermediate (2 or 3)	10 (55.6)
High-intermediate (4 or 5)	6 (33.3)
Clinical Staging - nodal lymphomas (n = 11) - n (%)	
I	2 (18.2)
II	2 (18.2)
III	0 (0.0)
IV	7 (63.6)
Gastrointestinal Staging (n = 7) - n (%)	
I	2 (28.6)
II1	3 (42.8)
II2	0 (0.0)
IIE	1 (14.3)
IV	1 (14.3)
LDH (U/L) – median (range)	200 (105–584)
Cell-of-origin - n (%)	
GC	7 (38.9)
ABC	8 (44.4)
NA	3 (16.7)

IPI (NCCN): International prognostic index (National Comprehensive Cancer Network); GC: germinal center; ABC: activate B-cell; NA: not available

### Response to treatment

Complete remission (CR) was achieved in 15 patients (83 %). One patient died from a respiratory infection before the end of treatment evaluation but was clinically in CR. Two patients (11 %) had primary refractory disease. Of the patients achieving CR, there was one early relapse. At the end of the study, five patients had died and the others remained in CR. The median time of observation between diagnosis and the last follow-up was 29.8 months (range: 7.5–61.0 months).

### ctDNA and PET scan

For the results of the PET scan at diagnosis (PET1), the median values for SUVmax, MTV, and TLG were, respectively: 32.84 (range: 4.3–45.0), 168.2 mL (range: 1.94–1654.5 mL), and 1936.4 (range: 6.1–11020.6). For the results of the PET scan after treatment (PET2), there was a significant decline in the median values of SUVmax, TMTV, and TLG (Table 2).

**Table 2:** Quantitative parameters of PET/CT image at diagnosis (PET1) and at the end of treatment (PET2).

	**PET 1**	**PET 2**	**Paired t-test**	**Δ (PET1–PET2)**
Patients - n	11	16		10
SUVmax median (range)	32.8 (4.3–45.1)	16.0 (2.9–57.9)	**0.127**	25.8 (−43.7 to 35.7)
TMTV (mL) median (range)	168.3 (1.9–1654.5)	7.5 (0–174)	**0.014**	433.8 (−84.2 to 1480.2)
TLG median (range)	1936.4 (6.1–11,020)	45.6 (0–2458)	**0.009**	2443.4 (−756 to 10,555)

SUVmax: Maximum standardized uptake volume; TMTV: Total metabolic tumor volume; TLG: Total lesion glycolysis

Correlations were found of ctDNA1 with TMTV (*r* = 0.51; *p*-value = 0.014) and TLG 2.5 (*r* = 0.47; *p*-value = 0.024) by PET at diagnosis. The volume of lesions with the highest SUV (Rd_volume) correlated positively and moderately with ctDNA1 (*r* = 0.57; *p*-value = 0.006). The TLG of this individual lesion (Rd_TLG) also correlated with ctDNA1 (*r* = 0.56; *p*-value = 0.02). There was also a correlation between ctDNA1 and radiomic features. The mean and range of the texture features that correlated with ctDNA1, as well as the respective correlation coefficients and *p*-values, are found in Table 3.

**Table 3:** Texture features with a significant correlation with circulating tumor DNA at diagnosis.

Texture feature	Median	Range	Correlation coefficient (r)	*p*-value
Homogeneity	0.60	(0.38–0.69)	0.61	0.02
Contrast	2.46	(1.27–10.43)	−0.68	0.01
SD of contrast	0.76	(0.39–3.64)	−0.62	0.019
Difference entropy	1.34	(1.09–1.76)	−0.61	0.02
Difference variance	1.05	(0.68–3.32)	−0.66	0.01
SD of difference variance	0.30	(0.18–1.42)	−0.62	0.01
SD of cluster prominence	34.63	(19.1–189.43)	−0.53	0.04
Measure of correlation 1	0.57	(0.28–0.67)	0.57	0.03

SD: Standard deviation

In the multiple regression only Rd_volume, Rd_TLG, homogeneity, difference entropy and measure of correlation participated in the model with R^2^ = 0.986. In a model using only parameters of the total tumor burden in PET imaging (TMTV, TLG and SUVmax, SUV min and SUV mean), only the TMTV remained in the model with R^2^ = 0.21. There was no correlation between the quantitative parameters of PET2 with the values of ctDNA2.

Ten patients had PET and ctDNA (measure in hGE/mL) data both at the diagnosis and at the end of the treatment. The Spearman correlation was calculated for these patients, which showed that ΔSUVmax (SUVmax1 – SUVmax2) strongly correlated with ΔctDNA (ctDNA1 – ctDNA2: r = −0.8788; *p*-value = 0.002).

### cfDNA analysis

Twelve (80 %) of the 15 CR patients had complete clearance of the mutation found in ctDNA1 by the end of the treatment. Concerning ctDNA quantification in hGE/mL, there was a decrease from ctDNA1 to ctDNA2 in 14 patients, 12 of whom achieved CR. One of the CR patients had an early relapse after eight months. The variation ranged from 0.18 % to 26.33 % (median 7.325 %). ctDNA2 was higher than ctDNA1 in four patients: one patient with disease progression and three CRs. The variation in these four cases ranged from 4.84 to 25.97 % (median: 9.835 %); at the time of the cfDNA2 collection, all four patients had mild active inflammatory/infectious processes. Supplementary Figure 2 shows the variations of ctDNA1 and ctDNA2, in log10 (hGE/mL). Supplemental Table 2 shows the correlation between clinical data and molecular response of the mutations attribute to the disease.

**Figure 1:**
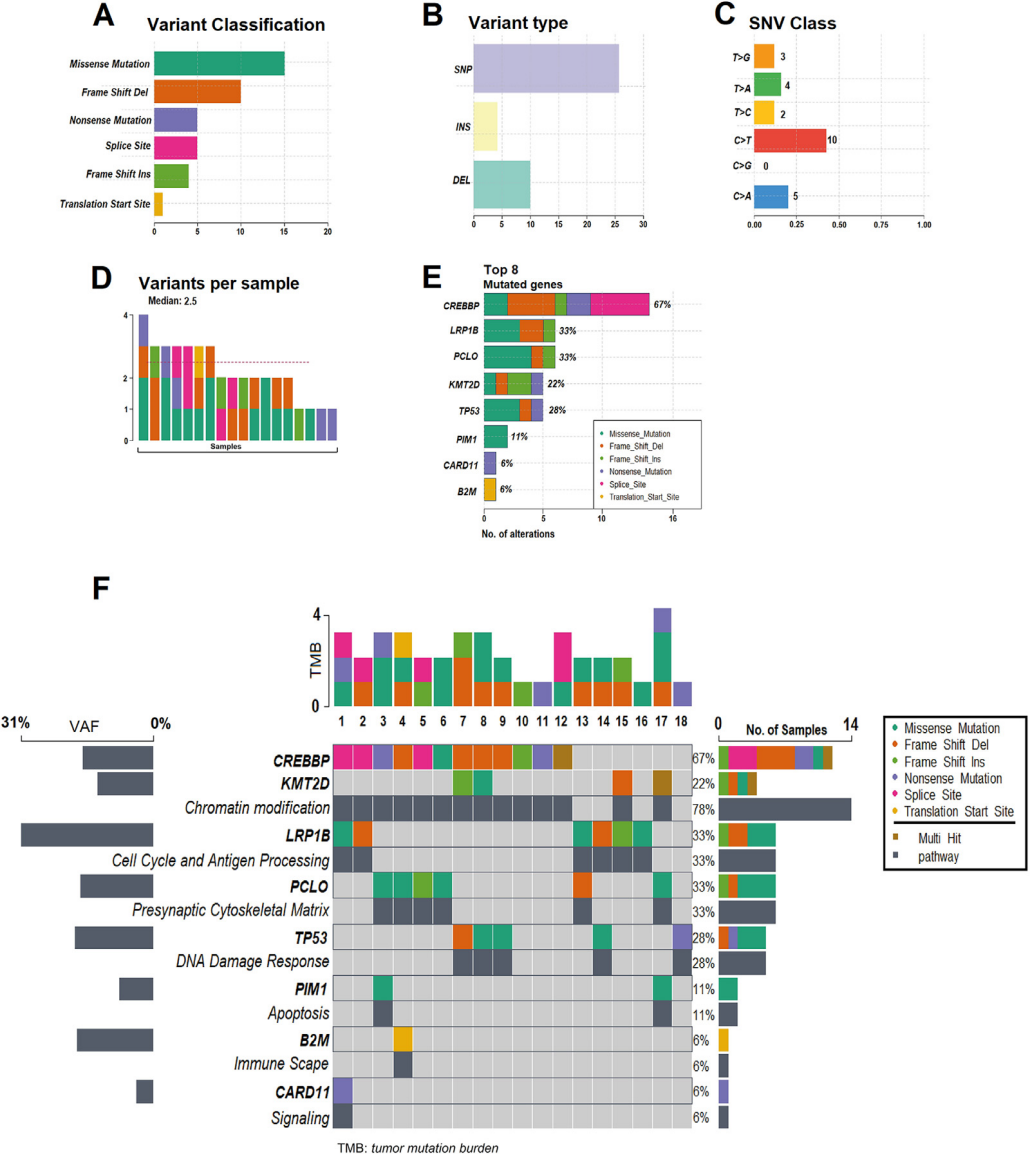
Summary of the mutations found in the 18 patients. (**A**) Variant classification, **(B)** Variant Type (single-nucleotide polymorphism [SNP] and indels), **(C)** Single nucleotide variant (SNV) class, **(D)** Variants per sample, (**E**) Top eight mutated genes. **(F)** Oncoplot showing the heatmap of the classification of variants for the eight main genes in 18 samples of the patients with DLBCL. The genes are ordered by frequency, (highest to lowest) mutated gene and identified according to variant classification (missense, frame shift ins/del, nonsense, splice site and translation start site). The mutated genes are grouped into the following functional categories: *CREBBP* and *KMT2D* (chromatin modification: 78 %), *LRP1B* (cell cycle and antigen processing: 33 %), *PCLO* (presynaptic cytoskeleton matrix: 33 %), *TP53* (DNA damage response: 28 %), *PIM1, B2M* and *CARD11* in functional pathways of apoptosis, immune escape and signaling: 6 % each). The average allele frequencies for each gene are displayed on the left. Variants noted as multi-hit are those identified in genes that undergo mutation more than once in the same sample from a patient.

**Figure 2:**
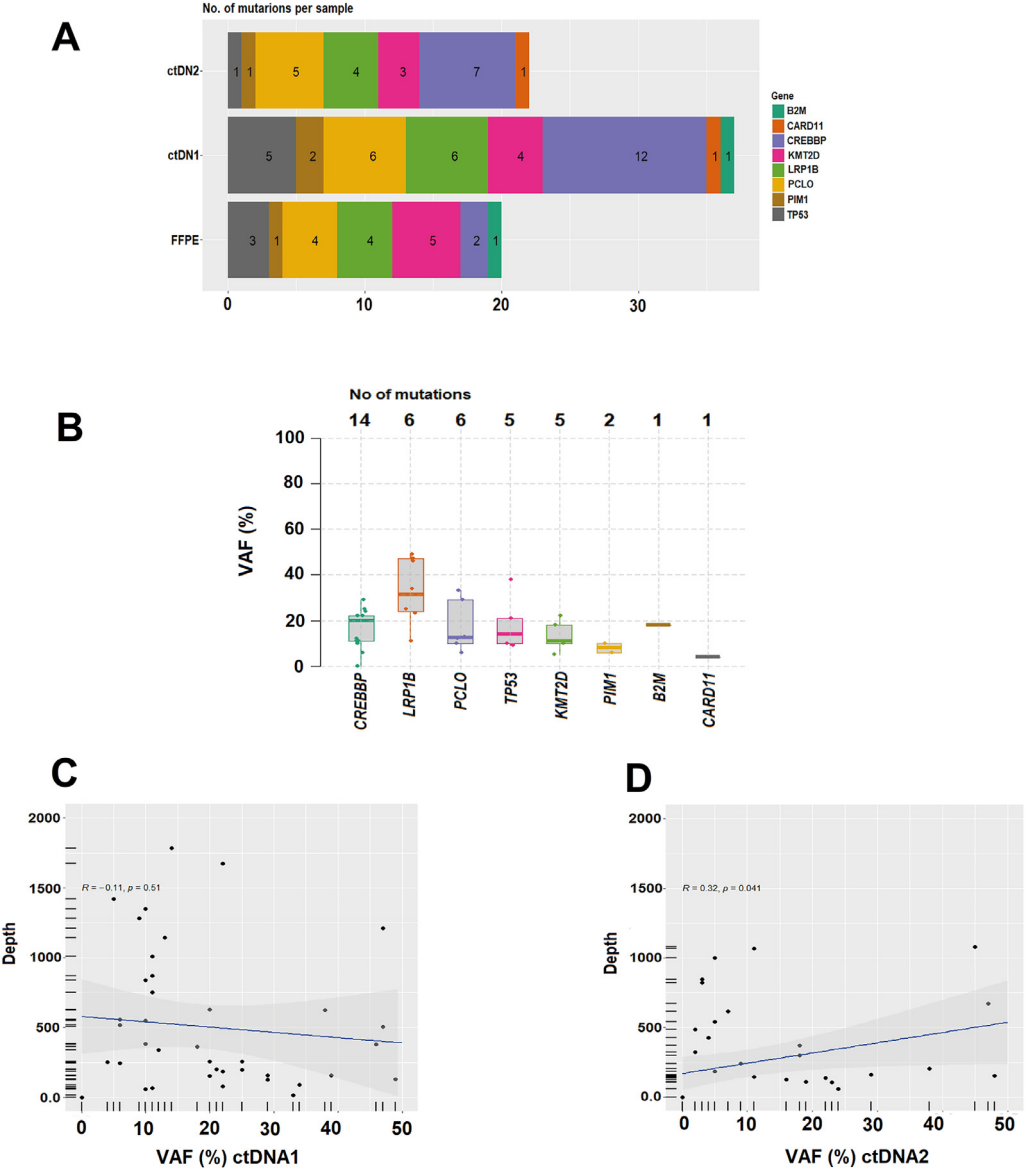
(A) Number of mutations per sample. (B) Median variant allele frequency (VAF) and top mutations identified. (C) and (D) Analysis of the linear correlation between the depth of NGS coverage and VAF in ctDNA1 and ctDNA2, respectively. (C) ctDNA1 and VAF (R = −0.11, *p*-value = 0.51), indicates a negative correlation between depth and VAF, and not statistically significant. (D) ctDNA2 and VAF (R = 0.32, *p*-value = 0.041), indicates a positive correlation with statistical difference (*p*-value <0.05).

In all 18 patients it was possible to detect at least one mutation (median: 2.5; range: 1–4) in the cfDNA1. By FFPE, seven cases did not show alterations. *CREBBP* (67 %) was the most frequent mutated gene, followed by *LRP1B* (33 %), *PCLO* (33 %), *TP53* (28 %), *KMT2D* (22 %), *PIM1* (11 %), *CARD11* (6 %) and *B2M* (6 %). Figure 1 and 2 summarize data on the mutations found in the 18 patients. Supplementary Figure 1 shows the mutations found in ctDNA1 and ctDNA2 with respective VAF changes for all 18 cases. Supplementary Tables 1 and 2 show clinical data with the mutations found in ctDNA1 and ctDNA2 (only considering the mutations most likely associated with the disease).

### ctDNA as clonal evolution

**Figure 3:**
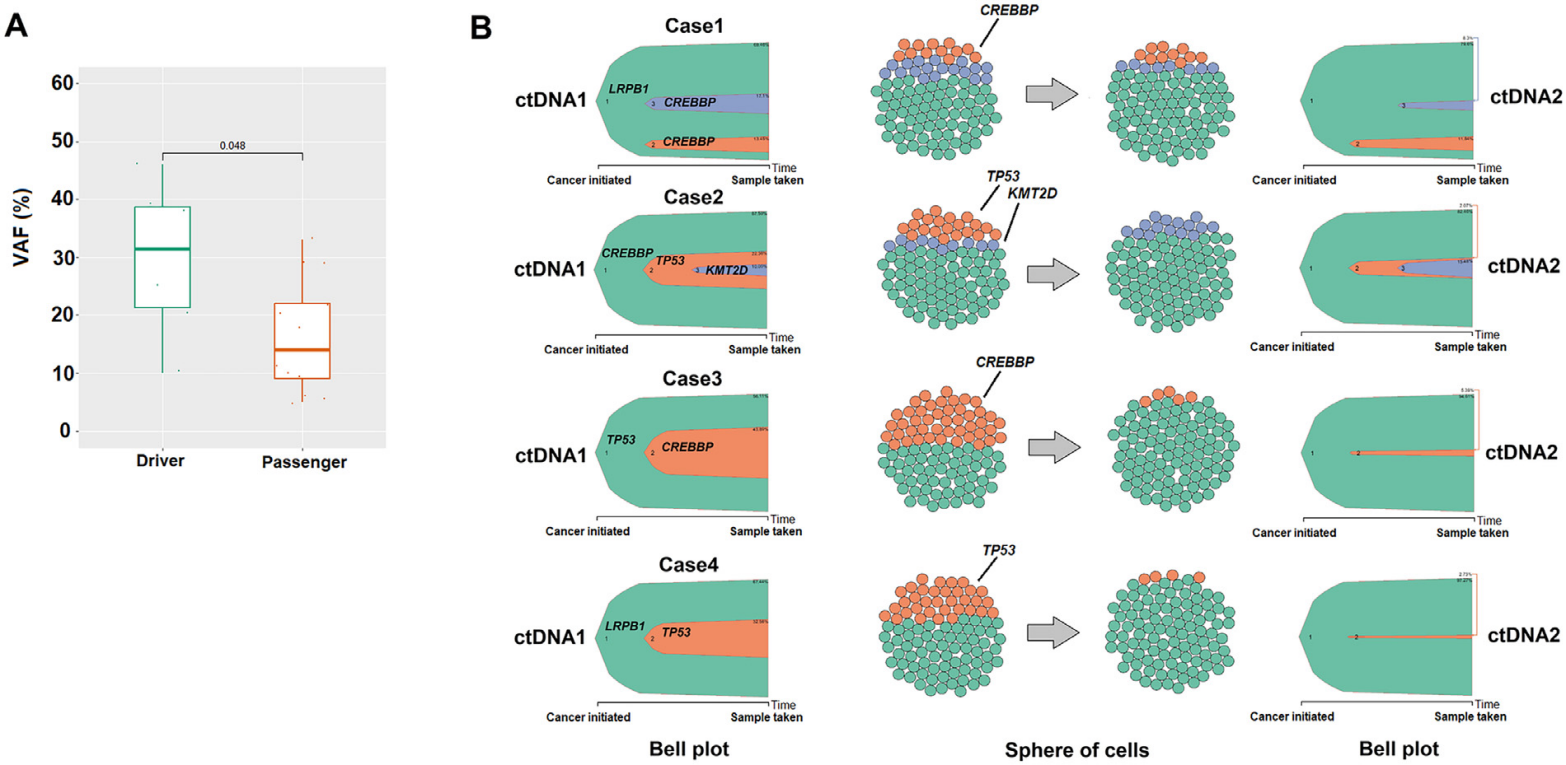
The result of clonal evolution analysis. (A) Comparison of the median variant allele frequency (VAF) between Driver (n = 8) and Passenger (n = 13) genes using the Wilcox test (*p*-value *=* 0.01). (B) Analysis of the clonal evolution of four cases (patients 3, 8, 7, and 14, see Table 2). The Bell plot and cell spheres show clonal dynamics over time and the proportion of clonal subpopulations, respectively in the two samples of circulating tumor DNA at diagnosis and after treatment. Cellular population parameters = 100 cell number.

A clonal evolution analysis was conducted in four patients (all in CR after treatment). We examined mutations identified in the founding clone and passenger mutations. The median number of occurrences in the founding clone was significantly higher (*p*-value = 0.048; Figure 3A). Clonal dynamics and the proportion of clonal subpopulations in ctDNA1 and ctDNA2 were estimated using the ClonEvol algorithm. A significant reduction in VAF was observed, particularly of the passenger mutations in ctDNA2 (Figure 3B). On the other hand, for the four patients analyzed, albeit in clinical remission, the driver mutations (genes *LRPB1, CREBBP*, and *TP53*) persisted in ctDNA2.

## Discussion

The analysis of ctDNA in lymphomas has been developed over recent years with the aim of genotyping and classifying the disease as well as of monitoring the response to treatment.^9^^,^^10^^,^^14^, ^15^, ^16^ It is still an evolving field. In this context, this study started in 2018 and customized a gene panel with a personalized cancer profiling strategy, using eleven genes that had been described as frequently mutated in DLBCL.^39^ The recruitment period for our patients was during the COVID-19 pandemic which affected the inclusion of new cases (there was only one patient included in 2020). This has also impacted the collection of cfDNA2, since 27 % of the patients died before completing the first line therapy. FFPE was available in 23/27 cases and in 7/18 no mutation could be detected. On the other hand, cfDNA1 had mutations detected in all 18 cases analyzed. Compared to the diagnostic tissue, ctDNA is readily available and can give more complete information on tumor genetics as it represents the spatial tumor heterogeneity.^15^

In the present study, cfDNA was collected before and after first-line treatment. Of the 18 patients analyzed, 14 had decreases in total ctDNA from diagnosis to the end of treatment. This was highly concordant with clinical response, since of these 14 patients only one showed refractory disease. All responding patients had at least two years of follow-up after treatment, which is an important landmark for DLBCL.^40^ On the other hand, this parameter was less reliable in the four cases where ctDNA2 was higher than ctDNA1. Three of these patients were in CR and one with disease progression. Considering the three CRs, at the time of the collection of cfDNA2 all of them had active but mild inflammatory processes. Elevated cfDNA quantification can happen in different situations not related to cancer, such as exercise, trauma, and inflammation.^41^, ^42^, ^43^ Any of these could have increased the value of ctDNA in these patients.

Clearance of specific somatic mutations in ctDNA has been correlated with clinical response in DLBCL patients after R-CHOP treatment^15^; this also happened in our patients. In the 15 patients in CR, 12 had complete clearance of the mutation (Supplementary Table 2).

A clonal evolution analysis was also performed in a subset of patients. cfDNA represents a potential test for clonal evolution in lymphoid neoplasms since it can capture the biological changes of the disease over time.^32^^,^^44^ The results of this study indicate a difference between the founding clone and subclones, with clonal selection due to treatment. Even with clinical remission, the driver mutations in the genes *LRPB1, CREBBP*, and *TP53* persisted in the ctDNA2 of the four patients analyzed. This could suggest the presence of clones resistant to treatment but without impact on the clinical disease since the patients are still in remission after more than two years. On the other hand, the subclones that reduced significantly after treatment could be more relevant to lymphomagenesis in these four cases. Using a more sensitive technique, with a greater number of patients and different sample collections over time may help to understand those patterns better.

This study has limitations. The limited number of genes used in the panel could have impaired our interpretation, since mutations linked to lymphomas could possibly not have been detected. However, the 11 genes used in the panel are among the most reported mutated genes in DLBCL according to different groups.^45^^,^^46^ We used an NGS-based strategy with personalized cancer profiling as previously described and validated in DLBCL.^10^^,^^15^^,^^16^^,^^18^ Nonetheless, this technique can have ‘false-negative’ results and there are currently new tests showing higher sensitivity.^47^ Paired analysis of DNA from leukocytes in peripheral blood could not be performed in all cases and, therefore, it is possible that some germline mutations were not correctly excluded. It was only possible to evaluate cfDNA twice, at diagnosis and at the end of treatment. Thus, a better follow-up of the kinetics of the disease of each patient was not possible. Finally, this study is unicentric and, although our center covers a large geographic area in the state of São Paulo, these results cannot be extrapolated to other geographic regions of Brazil.

To the best of our knowledge, this is the first study to evaluate ctDNA in DLBCL Brazilian patients. Our data show the utility of ctDNA as a non-invasive method to detect mutations associated with lymphoma and observe the changes at the end of treatment. Most patients in CR after treatment had decreases in ctDNA as measured in hGE/mL. This can be explored as another response assessment marker together with PET/CT. The combination of these tests is promising as has been reported by different group.^8^^,^^22^, ^23^, ^24^ The data of this study, although the number of patients is small, showed possible correlations with ctDNA quantification and PET/CT parameters. Hence, assessment of ctDNA as a marker of disease activity is a noninvasive technique that could be widely used if well standardized and validated.

The radiomic features of PET/CT were examined. These features are related to gray-tone spatial-dependence matrices that measure the internal variation of the signals and so indicate proliferation spots and necrotic areas. These features were highly correlated with the quantity of ctDNA at diagnosis. This analysis, which is beginning to be explored in imaging,^48^^,^^49^ could also help to identify tumor characteristics and their changes after treatment.

In conclusion, ctDNA has several utilities in patients with DLBCL. We believe that with a larger panel of recurrent mutated genes, with samples obtained at different collection points and a larger number of patients, it will be possible to draw more conclusions on the prognostic and decision-making value of this test.

## Author Contributions

Conceptualization GD, ENF, MLLC, ILM; methodology; GD, JHSM, FL, LGP, ENF, MEST, CDR, LF; software JHSM; validation GD, JHSM, FL, LGP, ENF; formal analysis JHSM, ILM; investigation GD, FL, LGP; resources JHSM, FL, LGP, ENF, FN, MLLC; data curation GD, JHSM, FL, LGP; writing—original draft preparation GD; writing, review and editing, GD, JHSM, FL, LGP, ENF, MEST, CDR, MLLC, ILM; visualization GD, JHSM; supervision MLLC, ILM; project administration GD, MLLC, ILM; funding acquisition MLLC, ILM All authors have read and agreed to the published version of the manuscript.

## Funding

This study had financial support from “Grupo Fleury”, Sao Paulo, SP, Brazil. I.L.M. had a grant from the National Research Council (“CNPq”, number 305110/2018-7).

The APC was funded by “Grupo de estudos multicêntricos em oncohematológicas” (GEMOH).

## Institutional Review Board Statement

The study was conducted according to the guidelines of the Declaration of Helsinki and approved by the institutional review board of the University of Campinas (UNICAMP), on May 17, 2018 (report number 2.659.716).

## Informed Consent Statement

Informed consent was obtained from all subjects involved in the study.

Data Availability Statement: The full data of this study is provided in the supplemental material. If any additional information is required, it can be requested to the corresponding author.

## Conflicts of interest

All authors declare that they have no conflict of interests.

## Appendix Supplementary materials

Image, image 1

Image, image 2

Image, application 3

Image, application 4

Image, application 5

## Footnotes

Supplementary material associated with this article can be found in the online version at doi:10.1016/j.htct.2024.07.005.
