# Supplementary figures and images for: Circulating tumor DNA in diffuse large B-cell lymphoma: analysis of response assessment, correlation with PET/CT and clone evolution
Source: Hematol Transfus Cell Ther. 2024 Sep 20;46(Suppl 6):S241–9. doi: 10.1016/j.htct.2024.07.005 (PMC11726095; doi:10.1016/j.htct.2024.07.005)

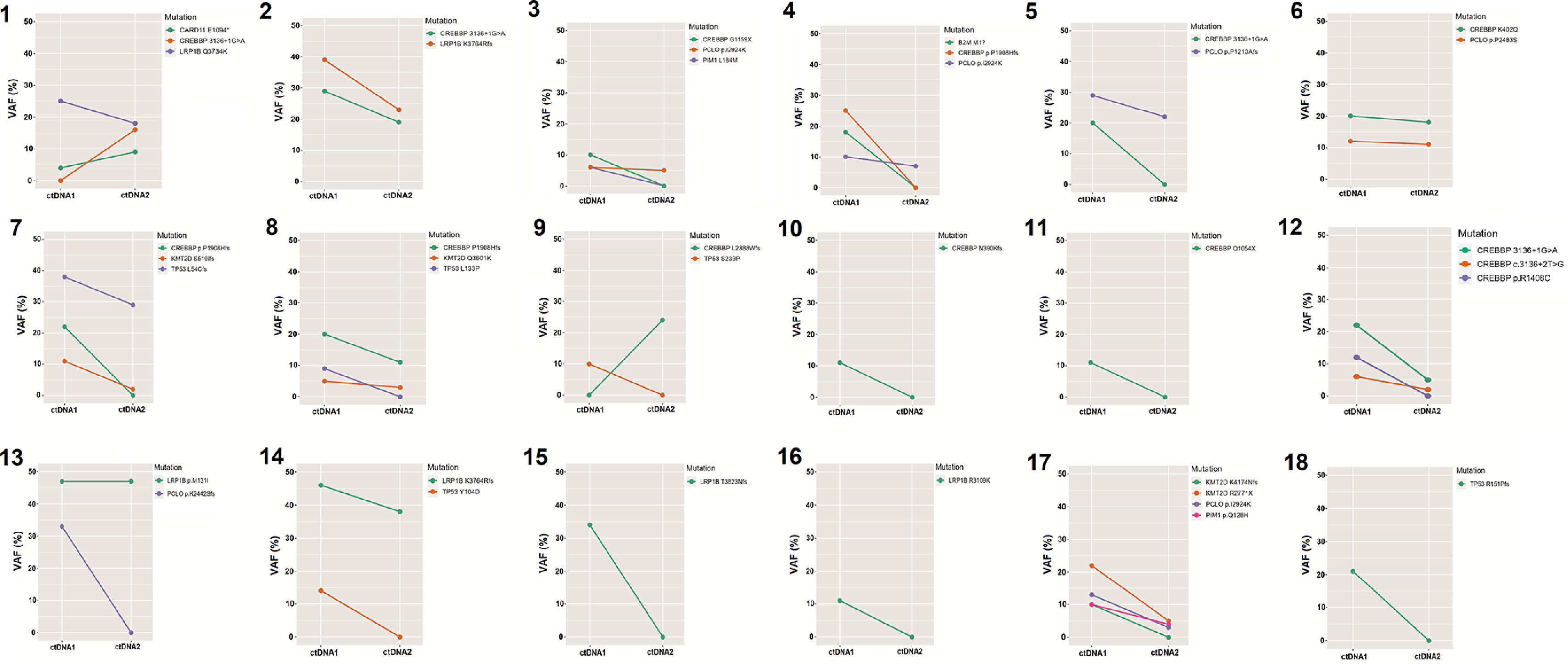

Supplement: Supplementary file 1 [file mmc1.jpg]

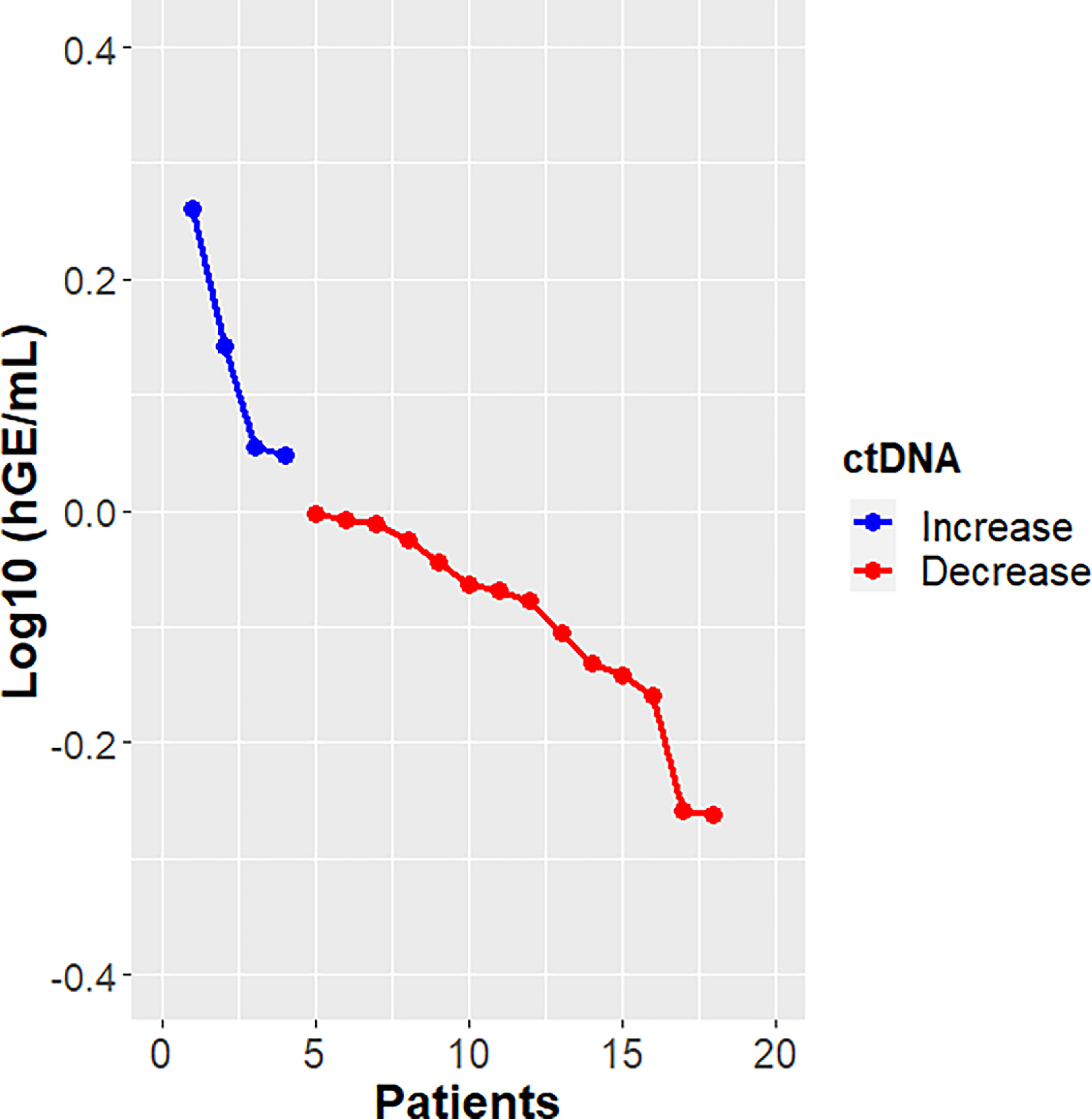

Supplement: Supplementary file 2 [file mmc2.jpg]
