# Supplementary material for: Circulating tumor DNA in diffuse large B-cell lymphoma: analysis of response assessment, correlation with PET/CT and clone evolution
Source: Hematol Transfus Cell Ther. 2024 Sep 20;46(Suppl 6):S241–9. doi: 10.1016/j.htct.2024.07.005 (PMC11726095; doi:10.1016/j.htct.2024.07.005)
Supplement: Supplementary file 3 [file mmc3.docx]

**SUPPLEMENTARY TEXT**

- **PET/CT**

The volumetric parameters Total Metabolic Tumor Volume (TMTV) and Total Lesion Glycolysis (TLG) were calculated from the initial whole-body ^18^F-FDG PET/CT images of the study subjects. The segmentation of the entire tumor volume was performed semi-automatically using the Beth Israel Plugin within the open-source software FIJI [1,2]. The semi-automatic segmentation comprised an initial fully automated phase using NifTI (Neuroimaging Informatics Technology Initiative) files with grown mask. The second part involved manual adjustments to exclude areas of normal physiological uptake that were included in the segmentation (false positives), and, when necessary, the manual inclusion of tumor areas that were excluded from the auto-segmentation. TMTV encompassed the entire tumor volume with a standardized uptake volume (SUV) above 2.5 [3]. TLG was obtained by multiplying TMTV by the average SUV of the segmented tumor volume [4]. Using the same software, measurements of radiomic features and their respective standard deviation (SD) were also collected for lesions with the highest SUV in the PET/CT images taken at the time of diagnosis. This tool is based on textural features extracted from the gray-tone spatial-dependence matrices proposed by Haralick et al [5].

- **Genomic Analysis**

Sequencing results were analyzed using Torrent Suite Software. Removal of low-quality reads, adapter trimming, and alignment of reads with the human reference genome (GRCh37-hg19) was done using the Torrent Mapping Alignment Program (TMAP). The variants were called Torrent Suite Variant (TSV) (version 5.8) [6].

The identified variants were recorded in a VCF (Variant Call Format) file, then annotated with ANNOVAR and InterVar [7,8] and filtered with the VarAFT tool [9], which included the following criteria: (i) changes causing amino acid exchange (Missense), (ii) changes in the gene reading frame (frameshift), (iii) causing stop codon (Nonsense) and (iv) all coding and flanking regions adjacent to exons and splice sites.

Variants with VAF (variant allele frequency) ≤ 50% were identified as heterozygous and considered for further analysis. Variants with read depth < 20 were excluded from the analysis, as were variants with allele frequency ≥ 1% in the healthy population [databases used include the 1000 Genomes Project, gnomAD [10,11], ABraOM (Brazilian archive of online mutations) [12] and minor allele frequency (MAF) < 1%)]. The remaining variants were evaluated with different functional impact algorithms of the deleterious potential of the mutations [13–17]. Variant deleterious effect was inferred through a combined annotation-dependent depletion (CADD) phred score of over 15 [18].

To avoid inclusion of possible germline mutations, we excluded alterations with VAF close to 50% and 100% when present in all 3 samples (FFPE, cfDNA1 and cfDNA2), mutations with report as polymorphisms in public genomic database (such as gnomad) and performed DNA extraction from leukocytes for comparison with the cfDNA (in 8 cases). Only mutations with VAF of ≥2% in cfDNA and ≥5% on tissue (FFPE) were considered.

Integrative Genomics Viewer (IGV) was used for visual data analysis, sequence alignments and filtering of possible sequencing artifacts [10]. The pathogenicity of the variants was identified according to the ACMG guidelines [19] using different algorithms. Variants of unknown significance were manually reviewed.

- **Exploring mutation data using Maftools**

The “Maftools” [20] was used to analyze somatic mutations (SNVs and small indels) that were stored in the form of Mutation Annotation Format (MAF) identified by sequencing the panel of 18 patients with DLBCL. The output format generated by ANNOVAR was converted into a MAF file with the “annovarToMaf” function implemented in R. The MAF file was used by the “read.maf” function to obtain a compatible MAF object for downstream analyses in Maftools.

- **Clone Evolution Analysis**

Clonal analysis was performed based on high confidence heterozygous mutation implemented in a hierarchical Bionomial beta mission model using the PyClone algorithm [21]. Somatic mutations with depth greater than 40x were used as input to PyClone. The minor copy number was used 0 and the major copy number as the total number of predicted copies. Total_copy_number PyClone beta-binomial model with the "total_copy_number" and option was run for 100,000 iterations for each case. Branch–based phylogenetic trees of the clones were constructed using the bootstrap resampling technique implemented in the ClonEvol algorithm [22] (R version 4.21). Mutations in indels, for which VAFs were poorly estimated, were not considered in the analysis.

**RESULTS OF cfDNA ANALYSIS**

In the 15 patients in CR, the mutation found in ctDNA1 had a complete clearance in ctDNA2 in 12 cases (80% of cases). Concerning ctDNA quantification in hGE/mL there was a decrease from ctDNA1 to ctDNA2 in 14 patients. Among them, 12 patients achieved CR after treatment. One of the CR patients had an early relapse after 8 months. The variation ranged from 0.18% to 26.33% (median 7.325%). ctDNA2 was higher than ctDNA1 in 4 patients: 1 patient with progression of disease (PD) and 3 CRs. The variation in these 4 cases ranges from 4.84% to 25.97% (mean 12.62% and median 9.835%). At the time of the cfDNA2 collection, all those 4 patients had mild active inflammatory/infectious process. Supplementary Figure 2 shows the variations of ctDNA1 and ctDNA2, in log10 (hGE/ml). Supplemental table 2 has the correlation between clinical data and molecular response of the mutations attribute to the disease.

In all 18 patients it was possible to detect at least one mutation (median=2.5, range 1 to 4) in the cfDNA1. There was a total of 37 different mutations. Concerning FFPE, 7 cases did not disclose alterations (a total of 20 mutations detected); 5 of those cases were limited-stage disease. The analysis of cfDNA2 detected 26 mutations. *CREBBP* (67%) was the most frequent mutated gene, followed by *LRP1B* (33%), *PCLO* (33%), *TP53* (28%), *KMT2D* (22%), *PIM1* (11%), *CARD11* (6%) and *B2M* (6%). There were 14 alterations in 12 patients in *CREBBP* (one patient had 3 different alterations – multi hit). Missense mutation was the predominant classification, with single-point mutation the most common one. Figure 1 is the summary of the mutations found in the 18 patients analyzed, with the most frequent 8 mutated genes, variant classification, variant type, and an oncoplot. The genes involved in chromatin modification (CREBBP and KMT2D) were the main changes identified in 78% of the cfDNA before treatment samples (figure 1F). Figure 2 shows the median variant allele frequency (VAF) of the top mutated genes, number of mutations per sample and correlation between the depth of NGS coverage and VAF in ctDNA1 and ctDNA2.

In ctDNA1, 12 patients had mutations on *CREBBP* – 4 patients with the same mutation (c.3136+1G>A) and 1 patient with 3 different mutations (multi hit). This was the most frequent mutated gene in cfDNA before treatment analysis. *TP53* was mutated in 5 patients, all in CR after treatment. The mutations were all different, 3 of them were missense, one frameshift and one stop-gain. In one case the mutation was present in all 3 samples (including FFPE), with VAF reduction from ctDNA1 to ctDNA2 (38% to 29%). In two cases the mutation was found in FFPE and cfDNA1 but not in cfDNA2. One of those patients had a second cancer 6 months after the RCHOP treatment and died of this disease. For the remaining two cases with *TP53* mutated, the mutations were only detected in ctDNA1. Supplementary Figure 1 shows the mutations found in ctDNA1 and ctDNA2, with respective VAF changes, of all 18 cases.

In 11 cases the same mutations were found in FFPE and cfDNA. In 7 cases no mutations were detected on FFPE. There was one patient with 2 alterations on FFPE where the second was not detected in cfDNA analysis (patient 15 – see supplemental material).

In 8 cases the same alteration found in FFPE and cfDNA1 was not detected in cfDNA2 (6 patients with CR – see supplemental material). There were no cases with alterations only in FFPE and cfDNA2. Supplementary Tables 1 and 2 shows clinical data with the mutations found in ctDNA1 and ctDNA2 (only considered the mutations most likely associated with the disease).

**References**

1. Schindelin J; Arganda-Carreras I; Frise E; Kaynig V; Longair M; Pietzsch T; Preibisch S; Rueden C; Saalfeld S; Schmid B; et al. Fiji: an open-source platform for biological-image analysis. *Nat Methods* **2012**, 9, 676-82.
2. Cypess A; Lehman S; Williams G; Tal I; Rodman D; Goldfine A; Kuo F; Palmer E; Tseng Y; Doria A; et al. Identification and importance of brown adipose tissue in adult humans. *N Engl J Med* **2009**, 360, 1509-17.
3. Takahashi M; Lorand-Metze I; Souza C; Mesquita C; Fernandes F; Carvalheira J; Ramos C. Metabolic Volume Measurements in Multiple Myeloma. *Metabolites* **2021** 11, 875.
4. McDonald J; Kessler M; Gardner M; Buros A; Ntambi J; Waheed S; van Rhee F; Zangari M; Heuck C; Petty N; et al. Assessment of total lesion glycolysis by 18F FDG PET/CT significantly improves prognostic value of GEP and ISS in myeloma. *Clinical Cancer Research* **2017**, 23, 1981-7.
5. Haralick RM; Shanmugam K; Dinstein IH. Textural Features for Image Classification. *IEEE* **1973**, SMC-3, 610-621.
6. Thermo Fisher Scientific: South San Francisco. Torrent Suite^TM^ Software 5.8 Release Notes (Pub. No. MAN0017478 Rev. A.0) [Internet]. 2018.
7. Chang X; Wang K. Wannovar: Annotating genetic variants for personal genomes via the web. J Med Genet. *BMJ Publishing Group Ltd* **2012**, *49*, 433–436. doi:10.1136/jmedgenet-2012-100918.
8. Li Q; Wang K. InterVar: Clinical Interpretation of Genetic Variants by the 2015 ACMG-AMP Guidelines. *Am J Hum Genet* **2017**,*100*, 267–280. doi:10.1016/j.ajhg.2017.01.004.
9. Desvignes JP; Bartoli M; Delague V; Krahn M; Miltgen M; Béroud C; Salgado D. VarAFT: A variant annotation and filtration system for human next generation sequencing data. *Nucleic Acids Res* **2018**, *46*, W545–W553. doi:10.1093/nar/gky471.
10. Lek M; Karczewski KJ; Minikel E; Samocha K; Banks E; Fennell T; O'Donnell-Luria A; Ware J; Hill A; Cummings B; et al. Analysis of protein-coding genetic variation in 60,706 humans. *Nature* **2016**, *536*, 285–291. doi:10.1038/NATURE19057.
11. Gudmundsson S; Singer-Berk M; Watts N; Phu W; Goodrich J; Solomonson M; Genome Aggregation Database Consortium; Rehm H; MacArthur D; O'Donnell-Luria A . Variant interpretation using population databases: lessons from gnomAD. *Hum Mutat* **2021**, *43*, 1012-1030. doi:10.1002/humu.24309.
12. Naslavsky M; Yamamoto G; de Almeida T; Ezquina S; Sunaga D; Pho N; Bozoklian D; Sandberg T; Brito L; Lazar M; et al. Exomic variants of an elderly cohort of Brazilians in the ABraOM database. *Hum Mutat* **2017**, *38*, 751–763. doi:10.1002/HUMU.23220.
13. Choi Y; Chan AP. PROVEAN web server: a tool to predict the functional effect of amino acid substitutions and indels. *Bioinformatics* **2015**, *31*, 2745–2747. doi:10.1093/BIOINFORMATICS/BTV195.
14. Ng P; Henikoff S. SIFT: predicting amino acid changes that affect protein function. *Nucleic Acids Res* **2003**, *31*, 3812. doi:10.1093/NAR/GKG509.
15. Adzhubei I; Schmidt S; Peshkin L; Ramensky V; Gerasimova A; Bork P; Kondrashov A; Sunyaev S. A method and server for predicting damaging missense mutations. *Nat Methods* **2010**, *7*, 248. doi:10.1038/NMETH0410-248.
16. Schwarz J; Cooper D; Schuelke M; Seelow D. MutationTaster2: mutation prediction for the deep-sequencing age. *Nat Methods* **2014**, *11*, 361–362. doi:10.1038/NMETH.2890.
17. Kircher M; Witten D; Jain P; O’roak B; Cooper G; Shendure J. A general framework for estimating the relative pathogenicity of human genetic variants. *Nat Genet* **2014**, *46*, 310–315. doi:10.1038/ng.2892.
18. Rentzsch P; Witten D; Cooper G; Shendure J; Kircher M. CADD: Predicting the deleteriousness of variants throughout the human genome. *Nucleic Acids Res* **2019**, *47*, D886–D894. doi:10.1093/nar/gky1016.
19. Richards S; Aziz N; Bale S; Bick D; Das S; Gastier-Foster J; Grody W; Hegde M; Lyon E; Spector E; et al. Standards and guidelines for the interpretation of sequence variants: A joint consensus recommendation of the American College of Medical Genetics and Genomics and the Association for Molecular Pathology. *Genet Med* **2015**, 17, 405–424.
20. Mayakonda A; Lin D; Assenov Y; Plass C; Koeffler H. Maftools: Efficient and comprehensive analysis of somatic variants in cancer. *Genome Res* **2018**, *28*, 1747–1756.
21. Roth A; Khattra J; Yap D; Wan A; Laks E; Biele J; Ha G; Aparicio S; Bouchard-Côté A; Shah S. PyClone: statistical inference of clonal population structure in cancer. *Nat Methods* **2014**, *11*, 396–398. doi:10.1038/nmeth.2883.
22. Dang H; White B; Foltz S; Miller C; Luo J; Fields R; Maher C. ClonEvol: clonal ordering and visualization in cancer sequencing. *Ann Oncol* **2017**, *28*, 3076–3082. doi:10.1093/ANNONC/MDX517.
