# Supplementary material for: Circulating tumor DNA in diffuse large B-cell lymphoma: analysis of response assessment, correlation with PET/CT and clone evolution
Source: Hematol Transfus Cell Ther. 2024 Sep 20;46(Suppl 6):S241–9. doi: 10.1016/j.htct.2024.07.005 (PMC11726095; doi:10.1016/j.htct.2024.07.005)
Supplement: Supplementary file 4 [file mmc4.docx]

Table 2. Clinical data, mutational status and treatment response. All the 18 patients are displayed on this table. The assigned mutation is the most likely associated with the patient’s lymphoma. Patient 2 responded to a second-line treatment and autologous transplant and is now in CR, ongoing. Patient 7 died of unrelated cause, in CR. Patient 10 died before the end of therapy PET and have no response status. CR = complete remission.

| **Patients** | **Age (y)** | **Clinical status** | **Mutation** | **VAF on ctDNA1 (%)** | **VAF on ctDNA2 (%)** | **Treatment response** |
| --- | --- | --- | --- | --- | --- | --- |
| Patient 2 | 65 | Gastric, stage IIE | *CARD11* | 4 | 9 | Primary refractory |
|  |  |  | *CREBBP* | 0 | 16 |  |
| Patient 4 | 58 | Gastric, stage I | *PIM1* | 6 | 0 | CR (ongoing) |
|  |  |  | *CREBBP* | 10 | 0 |  |
| Patient 5 | 73 | Gastric, stage II1 | *B2M* | 18 | 0 | CR (ongoing) |
|  |  |  | *CREBBP* | 25 | 0 |  |
| Patient 9 | 74 | Gastric, stage II1 | *TP53* | 10 | 0 | CR (ongoing) |
| Patient 13 | 48 | Gastric, stage II1 | *PCLO* | 33 | 0 | CR (ongoing) |
| Patient 14 | 51 | Gastric, stage IV (bone) | *TP53* | 14 | 0 | CR (ongoing) |
| Patient 16 | 63 | Gastric, stage I | *LRP1B* | 11 | 0 | CR (ongoing) |
| Patient 1 | 77 | Nodal (cervical), stage II | *CREBBP* | 20 | 0 | CR (ongoing) |
| Patient 3 | 59 | Nodal (cervical), stage I | *LRP1B* | 39 | 23 | CR (ongoing) |
|  |  |  | *CREBBP* | 29 | 19 |  |
| Patient 6 | 59 | Nodal, stage IV | *PCLO* | 12 | 11 | CR (ongoing) |
|  |  |  | *CREBBP* | 2 | 2 |  |
| Patient 7 | 58 | Skin and nodal, stage IV | *TP53* | 38 | 29 | CR |
|  |  |  | *CREBBP* | 22 | 0 |  |
|  |  |  | *KMT2D* | 11 | 2 |  |
| Patient 8 | 36 | Nodal (cervical and mediastinum), stage II | *TP53* | 9 | 0 | CR (ongoing) |
|  |  |  | *CREBBP* | 20 | 11 |  |
| Patient 10 | 84 | Nodal (inguinal), stage I | *CREBBP* | 11 | 0 |  |
| Patient 11 | 64 | Nodal, stage IV | *CREBBP* | 11 | 0 | CR (ongoing) |
| Patient 12 | 53 | Bone and nodal, stage IV | *CREBBP* | 12 | 0 | CR (ongoing) |
| Patient 15 | 66 | Nodal, stage IV | *LRP1B* | 34 | 0 | CR (early relapse - died due to the lymphoma) |
| Patient 17 | 24 | Nodal, stage IV (lung) | *KMT2D* | 22 | 5 | Primary refractory (died due to the lymphoma) |
| Patient 18 | 29 | Nodal, stage IV | *TP53* | 21 | 0 | CR (died due to a second cancer) |
