# Supplementary material for: Circulating tumor DNA in diffuse large B-cell lymphoma: analysis of response assessment, correlation with PET/CT and clone evolution
Source: Hematol Transfus Cell Ther. 2024 Sep 20;46(Suppl 6):S241–9. doi: 10.1016/j.htct.2024.07.005 (PMC11726095; doi:10.1016/j.htct.2024.07.005)
Supplement: Supplementary file 5 [file mmc5.docx]

**Table A1:**

| **ID** | **GENE** | **Transcript** | **Exon** | **HGVSc** | **HGVSp** | **Variant_Classification** | **ExonicFunc.refGene** | **ACMG Classifier** | **COSMIC ID** | **dbSNP** | **VAF-FFPE** | **VAF-cfDNA1** | **VAF-cfDNA2** |
| --- | --- | --- | --- | --- | --- | --- | --- | --- | --- | --- | --- | --- | --- |
| 1 | ***LRP1B*** | NM_018557 | exon73 | c.C11200A | p.Q3734K | Missense_Mutation | nonsynonymous SNV | Uncertain significance | COSV67187972 | rs35546150 | 0.22 | 0.25 | 0.18 |
|  | ***CARD11*** | NM_032415 | exon25 | c.3278dupC | p.E1094* | Nonsense_Mutation | stopgain | Likely pathogenic | . | . | 0.00 | 0.04 | 0.09 |
|  | ***CREBBP*** | NM_001079846 | exon15 | c.3136+1G>A | . | splicing_site | splicing | Likely pathogenic | . | . | 0.00 | 0.00 | 0.16 |
| 2 | ***LRP1B*** | NM_018557 | exon74 | c.11291delA | p.K3764Rfs*35 | Frame_Shift_Del | frameshift deletion | . | **.** | . | 0.65 | 0.39 | 0.23 |
|  | ***CREBBP*** | NM_001079846 | exon15 | c.3136+1G>A | . | splicing_site | splicing | Likely pathogenic | . | . | 0.00 | 0.29 | 0.19 |
| 3 | ***PIM1*** | NM_001243186 | exon4 | c.C550A | p.L184M | Missense_Mutation | nonsynonymous SNV | Uncertain significance |  | . | 0.37 | 0.06 | 0.00 |
|  | ***CREBBP*** | NM_001079846 | exon17 | c.G3475T | p.G1159X | Missense_Mutation | nonsynonymous SNV | Uncertain significance |  | . | 0.14 | 0.10 | 0.00 |
|  | ***PCLO*** | NM_014510 | exon5 | c.T8771A | p.I2924K | Missense_Mutation | nonsynonymous SNV | Uncertain significance | COSV61645633 | . | 0.13 | 0.06 | 0.05 |
| 4 | ***B2M*** | NM_004048 | exon1 | c.T2A | p.M1? | Translation_Start_Site | 5-UTR | Uncertain significance | COSV62563048 | . | 0.27 | 0.18 | 0.00 |
|  | ***CREBBP*** | NM_001079846 | exon30 | c.5723delC | p.P1908Hfs*30 | Frame_Shift_Del | frameshift deletion | Uncertain significance | . | rs587783507 | 0.00 | 0.25 | 0.00 |
|  | ***PCLO*** | NM_014510 | exon5 | c.T8771A | p.I2924K | Missense_Mutation | nonsynonymous SNV | Uncertain significance | COSV61645633 | . | 0.07 | 0.10 | 0.07 |
| 5 | ***CREBBP*** | NM_001079846 | exon15 | c.3136+1G>A | . | splicing_site | splicing | Likely pathogenic | . | . | 0.00 | 0.20 | 0.00 |
|  | ***PCLO*** | NM_014510 | exon4 | c.3635dupA | p.P1213Afs*4 | Frame_Shift_Ins | frameshift insertion | Likely pathogenic | COSV61645633 | rs376216629 | 0.00 | 0.29 | 0.22 |
| 6 | ***PCLO*** | NM_014510 | exon5 | c.C7447T | p.P2483S | Missense_Mutation | nonsynonymous SNV | Uncertain significance | COSV61648600 | . | 0.07 | 0.12 | 0.11 |
|  | ***CREBBP*** | NM_001079846 | exon4 | c.A1204C | p.K402Q | Missense_Mutation | nonsynonymous SNV | Uncertain significance | . | . | 0.00 | 0.20 | 0.18 |
| 7 | ***TP53*** | NM_001126118 | exon3 | c.160delC | p.L54Cfs*30 | Frame_Shift_Del | frameshift deletion | Likely pathogenic | . | . | 0.32 | 0.38 | 0.29 |
|  | ***CREBBP*** | NM_001079846 | exon30 | c.5723delC | p.P1908Hfs*30 | Frame_Shift_Del | frameshift deletion | Uncertain significance | . | rs587783507 | 0.17 | 0.22 | 0.00 |
|  | ***KMT2D*** | NM_003482 | exon10 | c.1526dupC | p.S510Ifs*24 | Frame_Shift_Ins | frameshift insertion | Likely pathogenic | . | . | 0.08 | 0.11 | 0.02 |
| 8 | ***TP53*** | NM_001126115 | exon4 | c.T398C | p.L133P | Missense_Mutation | nonsynonymous SNV | Pathogenic/Likely_pathogenic | COSV52732531 | . | 0.33 | 0.09 | 0.00 |
|  | ***KMT2D*** | NM_003482 | exon39 | c.C10801A | p.Q3601K | Missense_Mutation | nonsynonymous SNV | Uncertain significance | . | . | 0.06 | 0.05 | 0.03 |
|  | ***CREBBP*** | NM_001079846 | exon30 | c.5723delC | p.P1908Hfs*30 | Frame_Shift_Del | frameshift deletion | Likely pathogenic | . | rs587783507 | 0.00 | 0.20 | 0.11 |
| 9 | ***TP53*** | NM_001126115 | exon7 | c.T715C | p.S239P | Missense_Mutation | nonsynonymous SNV | Uncertain significance | COSV53556884 | . | 0.00 | 0.10 | 0.00 |
|  | ***CREBBP*** | NM_001079846 | exon30 | c.7162delC | p.L2388Wfs*10 | Frame_Shift_Del | frameshift deletion | Likely pathogenic | . | . | 0.00 | 0.00 | 0.24 |
| 10 | ***CREBBP*** | NM_001079846 | exon4 | c.1169dupA | p.N390Kfs*24 | Frame_Shift_Ins | frameshift insertion | . | . | . | 0.00 | 0.11 | 0.00 |
| 11 | ***CREBBP*** | NM_001079846 | exon16 | c.C3160T | p.Q1054X | Nonsense_Mutation | stopgain | Likely pathogenic | COSV52129403 | . | 0.00 | 0.11 | 0.00 |
| 12 | ***CREBBP*** | NM_001079846 | exon15 | c.3136+1G>A | . | splicing_site | splicing | Likely pathogenic | . | . | 0.00 | 0.22 | 0.05 |
|  | ***CREBBP*** | NM_001079846 | exon25 | c.C4222T | p.R1408C | Missense_Mutation | nonsynonymous SNV | Pathogenic | COSV52113365 | rs398124146 | 0.00 | 0.12 | 0.00 |
|  | ***CREBBP*** | NM_001079846 | exon15 | c.3136+2T>G | . | splicing_site | splicing | Likely pathogenic | . | . | 0.00 | 0.06 | 0.02 |
| 13 | ***LRP1B*** | NM_018557 | exon4 | c.G393A | p.M131I | Missense_Mutation | nonsynonymous SNV | Uncertain significance | COSV67226943 | rs151247276 | 0.62 | 0.47 | 0.47 |
|  | ***PCLO*** | NM_014510 | exon5 | c.7325delA | p.K2442Sfs*2 | Frame_Shift_Del | frameshift deletion | . | . | . | 0.38 | 0.33 | 0.00 |
| 14 | ***LRP1B*** | NM_018557 | exon74 | c.11291delA | p.K3764Rfs*35 | Frame_Shift_Del | frameshift deletion | Likely pathogenic | . | . | 0.00 | 0.46 | 0.38 |
|  | ***TP53*** | NM_001126115 | exon3 | c.T310G | p.Y104D | Missense_Mutation | nonsynonymous SNV | Pathogenic | COSV52672888 | rs587782289 | 0.00 | 0.14 | 0.00 |
| 15 | ***LRP1B*** | NM_018557 | exon75 | c.11467dupA | p.T3823Nfs*8 | Frame_Shift_Ins | frameshift insertion | Likely pathogenic | . | . | 0.53 | 0.34 | 0.00 |
|  | ***KMT2D*** | NM_003482 | exon34 | c.9265dupG | p.V3089Gfs*9 | Frame_Shift_Del | frameshift deletion | Pathogenic | . | . | 0.18 | 0.00 | 0.00 |
| 16 | ***LRP1B*** | NM_018557 | exon59 | c.G9326A | p.R3109K | Missense_Mutation | nonsynonymous SNV | Uncertain significance | . | . | 0.00 | 0.11 | 0.00 |
| 17 | ***KMT2D*** | NM_003482 | exon33 | c.C8311T | p.R2771X | Nonsense_Mutation | stopgain | Likely pathogenic | COSV56428815 | . | 0.24 | 0.22 | 0.05 |
|  | ***KMT2D*** | NM_003482 | exon39 | c.12522delA | p.K4174Nfs*41 | Frame_Shift_Del | frameshift deletion | Likely pathogenic | . | . | 0.20 | 0.10 | 0.00 |
|  | ***PIM1*** | NM_001243186 | exon2 | c.G384T | p.Q128H | Missense_Mutation | nonsynonymous SNV | Uncertain significance | COSV65166397 | . | 0.00 | 0.10 | 0.04 |
|  | ***PCLO*** | NM_014510 | exon5 | c.T8771A | p.I2924K | Missense_Mutation | nonsynonymous SNV | Uncertain significance | COSV61645633 | . | 0.00 | 0.13 | 0.03 |
| 18 | ***TP53*** | NM_001126115 | exon4 | c.451_452insCCGGCTAAGGGGT | p.R151Pfs*3 | Nonsense_Mutation | stopgain | Likely pathogenic | . | . | 0.14 | 0.21 | 0.00 |

* The accession number (NM_) is the National Center for Biotechnology Information database, Reference Sequence (https://www.ncbi.nlm.nih.gov/RefSeq); HGVSc, Human Genome Variation Society Transcript Nomenclature (for coding region); HGVSp, Human Genome Variation Society protein nomenclature; ACMG, The American College of Medical Genetics and Genomics; Pathogenic or Likely Pathogenic, VUS, variant of uncertain significance; VAF: Variant allele frequency is the fraction of the variant allele.

**This patients order is different than the used on Table A2 and the text.
